# Resistance of *Bt*-maize (MON810) against the stem borers *Busseola fusca* (Fuller) and *Chilo partellus* (Swinhoe) and its yield performance in Kenya

**DOI:** 10.1016/j.cropro.2016.07.023

**Published:** 2016-11

**Authors:** Tadele Tefera, Stephen Mugo, Murenga Mwimali, Bruce Anani, Regina Tende, Yoseph Beyene, Simon Gichuki, Sylvester O. Oikeh, Francis Nang'ayo, James Okeno, Evans Njeru, Kiru Pillay, Barbara Meisel, B.M. Prasanna

**Affiliations:** a International Maize and Wheat Improvement Center (CIMMYT), ICRAF House, UN Avenue, Gigiri, PO Box 1041, 00621, Village Market, Nairobi, Kenya; b International Center of Insect Physiology & Ecology (icipe), ILRI Campus, Gurd Shola, PO Box 5689, Addis Ababa, Ethiopia; c Kenya Agricultural and Livestock Research Organization (KALRO), 340-90100, Katumani, Machakos, Kenya; d Africa Agricultural Technology Foundation, P.O. Box 30709-00100, Nairobi, Kenya; e Monsanto Nairobi, P.O. Box 47686, Nairobi, Kenya; f Monsanto South Africa (Pty) Ltd, PO Box 69933, Bryanston, 2021, South Africa

**Keywords:** *Bt*-maize, *Busseola fusca*, *Chilo partellus*, Confined field trial, GM crops, MON810

## Abstract

A study was conducted to assess the performance of maize hybrids with *Bt* event MON810 (*Bt*-hybrids) against the maize stem borer *Busseola fusca* (Fuller) in a biosafety greenhouse (BGH) and against the spotted stem borer *Chilo partellus* (Swinhoe) under confined field trials (CFT) in Kenya for three seasons during 2013–2014. The study comprised 14 non-commercialized hybrids (seven pairs of near-isogenic *Bt* and non-*Bt* hybrids) and four non-Bt commercial hybrids. Each plant was artificially infested twice with 10 first instar larvae. In CFT, plants were infested with *C. partellus* 14 and 24 days after planting; in BGH, plants were infested with *B. fusca* 21 and 31 days after planting. In CFT, the seven *Bt* hybrids significantly differed from their non-Bt counterparts for leaf damage, number of exit holes, percent tunnel length, and grain yield. When averaged over three seasons, *Bt*-hybrids gave the highest grain yield (9.7 t ha^−1^), followed by non-*Bt* hybrids (6.9 t ha^−1^) and commercial checks (6 t ha^−1^). *Bt*-hybrids had the least number of exit holes and percent tunnel length in all the seasons as compared to the non-*Bt* hybrids and commercial checks. In BGH trials, *Bt*-hybrids consistently suffered less leaf damage than their non-Bt near isolines. The study demonstrated that MON810 was effective in controlling *B. fusca* and *C. partellus*. *Bt*-maize, therefore, has great potential to reduce the risk of maize grain losses in Africa due to stem borers, and will enable the smallholder farmers to produce high-quality grain with increased yield, reduced insecticide inputs, and improved food security.

## Highlights

• The present trials were conducted to generate empirical information for commercialization of Bt-maize in Kenya.
• This study reports on the efficacy of Bt maize (MON810) against the tem borers, *Busseola fusca* and *Chilo partellus* in Kenya.
• *Bt-* maize significantly reduced leaf damage, number of exit holes, percent tunnel length, and increased grain yield.

## 1 Introduction

Maize is currently cultivated in about 25 million ha in sub-Saharan Africa, largely in smallholder systems that produce 38 million metric tons, primarily for food. An additional 2.8 million ha is grown in South Africa, mostly in large-scale commercial production, much of it for animal feed (Smale et al., 2011). The highest amounts of maize consumed are found in Southern Africa at 85 kg/capita/year as compared to 27 in East Africa and 25 in West and Central Africa. In Lesotho, Malawi, South Africa, Zambia and Zimbabwe, average consumption is over 100 kg/capita/year. These amounts represent more than 50% of total calories in Lesotho, Malawi and Zambia, 43% in Zimbabwe, and 31% in South Africa (Shiferaw et al., 2011).

Average national yields for maize in the main producing countries of the eastern and southern Africa are reported to range between 1.1 tons and 1.8 tons per hectare, although they have occasionally surpassed 2–3 tons per hectare in Zambia, Zimbabwe, Kenya and Ethiopia (Smale et al., 2011). Limited access to improved inputs (including improved maize varieties and fertilizers), recurrent drought, poor soil fertility, diseases, weeds and insect pests are major factors contributing to the low maize yields in sub-Saharan Africa (Smale et al., 2011, Shiferaw et al., 2011).

Lepidopteran stem borers cause significant negative impact on maize yields in Africa due to their damage to the leaves, stem and ears. The spotted stem borer *Chilo partellus* (Swinhoe) (Crambidae) and the African stem borer *Busseola fusca* (Fuller) (Noctuidae) are the two most economically important stem borers of maize. A study made in 2000–2001 in Kenya, estimated annual maize yield lost due to stem borers damage was on average 14%, about 0.44 million tons valued US$ 25–60 million, which was enough to feed 3.5 million people per annum at per capita maize consumption of 125 kg (Odendo et al., 2003).

Application of chemical insecticides has been recommended to protect maize crop against stem borers. However, insecticides are too expensive for subsistence farmers in most parts of Africa, besides posing environmental and health hazards if not used judiciously or with proper safety measures (PAN UK, 2003). Therefore, effective and economically feasible stem borer control practices need to be made available to farmers in sub-Saharan Africa.

*Bt* maize has been genetically modified to produce a Bt protein to control maize insect pests including European corn borer (*Ostrinia nubilalis* Hübner) and southwestern corn borer (*Diatraea grandiosella* Dyar) (Hutchinson et al., 2010); the gene that produces this insecticide is transferred to maize from a soil bacterium *Bacillus thuringiensis* (Saxena and Stotzky, 2000). *Bt* maize has the potential to increase yields where stem borers are a major constraint (Van Wyk et al., 2008, Tende et al., 2010). South Africa is the only country in Africa where farmers grow Bt maize (Assefa and Van Den Berg, 2009). In 2014, 2.14 million hectares of the total maize area in South Africa was Bt maize (James, 2014). *Bt*-maize is extensively cultivated in the USA, Argentina, Canada, and recently in the Philippines. Worldwide, the total area planted with genetically modified maize is 57 million hectares (James, 2013).

A number of commercial *Bt* formulations have been used for a long time as effective biopesticides for the protection of food crops, ornamentals, forest trees and stored grains against insect-pests (Meadows, 1993). *Bt* formulations are highly specific, harmless to humans, animals, and a wide array of non-target pests (Saxena and Stotzky, 2000). Therefore, it is ideally suited for integrated pest management (IPM) strategies (Nester et al., 2002). In spite of these advantages, *Bt* based formulations have several disadvantages, as they need to be applied repeatedly and are effective only against immature stages of target insects feeding on exposed plant surfaces (McGaughey and Whalon, 1992). These limitations can be overcome by expression of *Bt*-proteins (endotoxins) in transgenic plants (Krattiger, 1997). *Bt-*maize has revolutionized stem borer control in several countries and also enabled growers to expand maize production into regions where high pest populations have made growing maize unprofitable (Hellmich and Hellmich, 2012). South Africa is the only country where *Bt*-maize is commercially grown in Africa (James, 2007). Growers are interested in *Bt*-crops because of the reduced need for application of broad-spectrum insecticides (Carpenter et al., 2002), increased or protected yields due to season-long control of the target insect pest (Rice and Pilcher, 1998), and improved grain quality as a result of lower mycotoxin levels due to reduction in fungal pathogens associated with insects feeding on the maize (Munkvold et al., 1999).

The National Biosafety Authority (NBA) of Kenya was established by the Biosafety Act No. 2 of 2009 to exercise general supervision and control over the transfer, handling and use of genetically modified organisms (GMOs). The present trials were conducted for three seasons under the supervision of the NBA to generate empirical information to guide decision making on application for approval to commercialize *Bt*-maize in Kenya. This study, therefore, reports on the efficacy of maize with event MON810 against the two major stem borers of maize, *B. fusca* and *C. partellus* in biosafety greenhouse and confined field trials, respectively, in Kenya.

## 2 Materials and methods

### 2.1 Germplasm

A set of 18 hybrids were used in this study, with seven pairs of near isolines of Bt (with event MON810 expressing Cry1Ab) and non-Bt hybrids, and four non-*Bt* commercial hybrids (KH 414–3, H516, WH505, DK8053). The *Bt*-hybrids and their non-*Bt* counterparts were sourced from Monsanto Company, and the non-*Bt* commercial hybrids were procured from a local market in Nairobi (Table 1).

### 2.2 Evaluation of *Bt*-maize against *C. partellus* under confined field trials

Confined field trials (CFT) were conducted at the Kenya Agricultural and Livestock Research Organization (KALRO), Maize Research Station in Kiboko, Makueni County, Kenya. Kiboko is located at 02°15′ S and 37°75′ E, and an elevation of 975 m above sea level (m.a.s.l.). The experimental site has sandy clay soils and receives on an average 530 mm annual rainfall, spread over two very short rainy seasons. The site had similar relative humidity and temperature during the study period. The site received high mean temperature (25–28 °C) from February to April and low temperature (20–21 °C) in July and August. The weather conditions were optimum for both maize and insect growth and development.

#### 2.2.1 Experimental design and management

The experiment was laid out in a randomized complete block design with four replications. Two seeds were sown per hill in a row of 5 m long and thinned to one seedling per hill 2 weeks after emergence. There were two rows per plot. The row-to-row distance was 0.75 m while plant-to-plant distance was 0.25 m, giving a plant density of 53,333 plants ha^−1^. Standard rates of fertilizers were applied (60 kg N and 60 kg P_2_O_5_ ha^−1^). Top dressing was done using nitrogen fertilizer in two splits. Supplemental irrigation was applied when needed. The fields were kept weed-free by hand weeding. There were a total of 21 plants per row as described above. The trial was repeated for three consecutive seasons; CFT-1 was planted in January 2013, CFT-2 in August 2013 and CFT-3 in May 2014.

#### 2.2.2 Artificial infestation with *C. partellus*

First instar larvae of *C. partellus* were obtained from the CIMMYT and KALRO, Katumani Stem Borer Mass Rearing Laboratory. Each row was divided using a string into two equal halves, one infested and one uninfested, excluding one border plant from both ends. A total of 20 plants per plot, 10 plants in the front half of each of the two rows, were infested artificially with ten *C. partellus* neonates per plant 14 and 24 days after planting using a camel hair brush.

#### 2.2.3 Data collection

Foliar damage for stem borer was assessed from the 20 artificially infested plants per plot, using a 1–9 scale; where 1= no visible damage; and 9 = completely damaged (Tefera et al., 2011). Foliar damage was first assessed 24 days after planting (V2 stage) just before the second round of artificial infestation was done on the same day. Foliar damage was also assessed 39 days after planting (V4 stage).

At physiological maturity (at harvest), the number of stem borer exit holes per plant (holes resulting from larval feeding which serve as exit holes for the moth) was counted from the 20 infested plants per plot. Each tunnel length per stem was measured after splitting the stems of the infested plants. Cumulative tunnel length was summed per plant. The cumulative tunnel length was expressed as percentage of total stem height. No larvae or pupae were recovered at harvest after splitting the stems. The infested plants were harvested excluding one border plant from both ends. Ears from each of these plants per plot were shelled separately, and grain weight taken at moisture content of 12.5–13%, and converted to grain yield per hectare.

### 2.3 Greenhouse evaluation of *Bt* maize against *B. fusca*

CFT was not conducted for *B. fusca* at Kiboko, Kenya, because the site is hot and dry, and does not represent an ideal habitat for *B. fusca*, a pest which inhabits and causes damage to cereal crops in cooler areas of Kenyan highlands (Muyekho et al., 2005). Preliminary unpublished observations from artificial infestation of maize with *B. fusca* larvae at Kiboko resulted in high larval mortality shortly after infestation; hence, greenhouse trials were designed. The *Bt*-maize, non-*Bt* maize, and non-*Bt* commercial hybrids described above were sown in the biosafety level-two greenhouse at KALRO, Nairobi. Three seeds were planted per plastic pot of 10 L (30 × 33 cm), filled with about six kg of sterilized soil; the pots were watered as required, and kept weed-free. The pots were arranged in completely randomized design; each pot was used as a replicate. Each plant was artificially infested twice with ten neonates per plant 21 and 31 days after planting using a camel hair brush. Leaf damage was assessed twice, 31 and 41 days after planting using the standard 1–9 visual scoring scale described above. The insect succumbed to early mortality before making damage to stems and only leaf damage was subject to analysis.

### 2.4 Statistical analysis

For the CFT data, percent tunnel length was angular transformed (arcsine √ proportion) in order to normalize the variance before analysis of variance (ANOVA). Number of exit holes and yield were not transformed. Foliar damage was categorical data and subjected to Kruskall-Wallis non-parametric analyses. Comparisons were made using rank sums to determine significant differences between means at *P* ≤ 0.05 (Dunn, 1964). For yield, number of exit holes and percent tunnel length, a two-sample *t*-test (Snedecor and Cochran, 1989) was used to determine the difference between *Bt* and non-Bt hybrids. A two way factorial analysis (with entry and season as fixed effects) was executed to determine interactions and main effects amongst the three sets of hybrids (averages of *Bt* hybrids, non-*Bt* near isogenic hybrids and non-*Bt* commercial checks) averaged across season. The means were separated using the least significance difference (LSD) at P = 0.05. Data were analyzed with Statistical Analysis Software (SAS, 2003). Untransformed (original) data were presented in results for tunnel length and foliar damage. For the biosafety greenhouse trial, leaf damage data by *B. fusca* were analyzed using Kruskall-Wallis non-parametric analyses.

## 3 Results

### 3.1 Effect of *Bt* maize on leaf, stem damage, grain yield under *C. partellus* infestation

Significant differences were mostly observed in leaf damage between entries and seasons; however, for damage 1, the damage were more significant in the second and third season than in the first season (Table 2). There were significant differences between entries in number of exit holes per plant in the first and third season; entry 1 and 2, entry 9 and 10, and entry 13 and 14 were consistently different among the seasons (Table 3). Per cent tunnel length significantly differed among seasons and in all entries except for entry 9 and 10, and entry 13 and 14 in the first season (Table 4). Significant differences were observed in yield between entry 1 and 2 in the three seasons (Table 5); however, majority of the entries showed significant differences in the second and third season than in the first season.

There were significant effects of hybrids, seasons and their interaction on grain yield (hybrids: *F*_*2, 27*_ = 142.2; P < 0.0001; seasons: *F*_*2, 27*_ = 102.1; P < 0.0001; hybrids by seasons: *F*_*4, 27*_ = 24.1; P < 0.0001) (Table 6). Number of exit holes were significantly affected by hybrids and seasons; however, the interaction between hybrids and seasons was not significant (hybrids: *F*_*2,27*_ = 37.2; *P* = P < 0.0001; seasons: *F*_*2,27*_ = 28.3; P < 0.0001; hybrids by seasons: *F*_*4,27*_ = 0.8; *P* = 0.53). Similarly, significant differences were also observed in tunnel length between hybrids and seasons but not in the interaction between hybrids and season (hybrids: *F*_*2,27*_ = 88.8; P < 0.0001; seasons: *F*_*2,27*_ = 4.9; P < 0.0001; hybrids by seasons: *F*_*4,27*_ = 1.7; *P* = 0.17) (Table 6). The significant interaction for yield can be explained by a lack of entry effect in season 1, whereas the Bt hybrids had higher yields than the non-Bt hybrids and commercial checks in seasons 2 and 3. When averaged over three seasons, *Bt*-hybrids gave the highest grain yield (9.7 t ha^−1^), followed by non-*Bt* hybrids (6.9 t ha^−1^) and commercial checks (6 t ha^−1^) (Table 6). *Bt*-hybrids had the least number of exit holes and percent tunnel length in all the seasons as compared to the non-*Bt* hybrids and commercial checks. The highest grain yield of 8.4 t ha^−1^ was obtained in third season and the least grain yield of about 5.8 t ha^−1^ was recorded in the first season (Table 6). The least number of exit holes and percent stem tunneled were recorded in third season.

### 3.2 Effect of *Bt* maize on leaf damage under *B. fusca* infestation

There were significant differences in leaf damage between *Bt*-hybrids and their near isogenic versions except for hybrids 1 and 2 (Table 7). *Bt*-hybrids consistently suffered less leaf damage than their near isogenic versions.

## 4 Discussion

The present study demonstrated the efficacy of MON810 in controlling the two major species of stem borers in Kenya, *B. fusca* and *C. partellus*. *Bt* maize was initially developed for the control of two stem borers in North America, namely *Ostrinia nubilalis* (Hubner) (Lepidoptera: Crambidae) (Ostlie et al., 1997) and *Diatraea grandiosella* (Dyar) (Lepidoptera: Crambidae) (Archer et al., 2001) before it was introduced for control of *B. fusca* (Fuller) (Lepidoptera: Noctuidae) and *C. partellus* (Swinhoe) (Lepidoptera: Crambidae) in South Africa (Gouse et al., 2005).

After splitting the stems, no larvae of *C. partellus* or *B. fusca* were recovered in the present study from Bt maize. Although the *Bt* maize that is used in South Africa effectively controls *B. fusca*, survival of this insect species on certain plant parts has been reported (Van Rensburg, 1998, Van Rensburg, 2001). The MON810 event is, however, reported to cause 100% mortality of *C. partellus* (Van Rensburg, 1998, Singh et al., 2005) and the pink stem borer, *Sesamia calamistis* (Hampson) (Lepidoptera: Noctuidae) (Van Wyk et al., 2007). Laboratory studies had earlier indicated that *C. partellus* and *B. fusca* larval survival and weight were lower after feeding on *Bt*-maize (Tende et al., 2010).

The *Bt*-hybrids had less leaf damage, number of exit holes and percent tunnel length compared to the commercial checks. This can likely be attributed to expression of the Bt toxin in leaves and stems. Castro (2002) reported protein expression for MON810 in all maize plant tissue. Different promoters have been used in various commercial *Bt*-maize hybrids and these different hybrids have been shown to express different amounts of toxin in different plant tissues (Van Wyk et al., 2009, Dutton et al., 2003). Cry1Ab protein expression in transgenic maize varieties containing the cauliflower mosaic virus (CaMV) 35 S promoter for MON810 and Bt11 expresses the toxin throughout the season in leaves, stem, roots, and kernels (EPA, 2000). Important behavioural implications may arise if differences in *Bt*-toxin concentrations exist within the plant. For example, if the larvae feed on silks and kernels with a lower toxin concentration, and only then penetrate the stems as 3rd instars, they may be able to survive inside the stems. Van Rensburg (2001) observed that protein expression was high enough during the vegetative stages of plant development when larvae feed only on leaf and stem tissue, but *B. fusca* 1st instars survived when fed on maize silks.

The *Bt*-hybrids in the present study gave the highest mean grain yield, 9.7 t ha^−1^, as compared to the mean grain yield of the non-Bt near isolines, 6.9 t ha^−1^, i.e. 28% yield advantage. Safeguarding maize yield through stem borer control with *Bt*-event will have a huge yield benefit to both smallholder and large-scale commercial maize farmers in Kenya and elsewhere in SSA. *Bt*-crops are particularly suitable for small-scale farmers since no equipment and pesticide knowledge are needed and these crops can reduce exposure of farmers to insecticides, especially for those using hand sprayers (Heldt, 2006, Qaim and de Janvry, 2005). Although we did not test this in the current study, another potential benefit of *Bt*-maize could be lesser accumulation of mycotoxins from opportunistic fungi that infect damaged ears (Munkvold et al., 1999). Healthier ears without insect-pest damage are less likely to be infected by fungi, which produce mycotoxins that are harmful, and often lethal, to humans and livestock (Miller et al., 2003).

Although MON810 has proved to be effective in the present study in controlling the stem borers, it is prudent to design insect-pest resistant management strategy at the outset for deploying *Bt*-maize for possible commercial cultivation in Kenya. Resistance has already been reported for some insect-pests. Fall armyworm, *Spodoptera frugiperda*, showed resistance to Cry1F in maize in Puerto Rico (Matten et al., 2008) and resistance of the stem borer *B. fusca* to *Bt* maize was reported by Van Rensburg (2007), six years after *Bt*-maize was introduced in South Africa. Different Cry genes target different receptors in the target insects, and therefore, require multiple mutations for resistance to develop in the insects (Zhao et al., 2003). Unlike South Africa where *Bt*-maize is mainly grown by large-scale commercial farmers, in the rest of African countries, maize is predominantly grown by subsistence farmers under diverse and genetically non-uniform farming practices which may play a role in delaying the evolution of resistance (Mulaa et al., 2007, Mulaa et al., 2011). In Africa, the target lepidopteran stem borers attack a wide range of wild grass species as well as cultivated cereal crops. Although abundance of stem borers are generally low on alternate hosts, wild grasses often are found in the vicinity of maize and other cereal fields, and may provide a refuge if *Bt* maize is introduced into the farming systems (Mulaa et al., 2011). The refuge component of the strategy requires that non-Bt crops are available in the cropping system, or nearby, so that susceptible individuals (ss) survive to mate with any resistant homozygous individuals (*R/R*) surviving on the *Bt* crops (Gould and Tabashnik, 1998).

Growing *Bt*-maize has been deemed compatible with other control methods, including integrated pest management (IPM) programs (Pons et al., 2005, Cotter, 2009). Compared to other IPM practices, growing *Bt-*maize is not knowledge-intensive because the technology is in the seed. This should be attractive to the smallholder maize farmers in Kenya or elsewhere in Africa, where poor infrastructure and inadequate extension services sometimes limit the use of conventional IPM practices. Maize farmers in African countries, however, often have other agronomic and socio-economic constraints besides pest management before deciding to adopt/grow *Bt* maize. Nevertheless, *Bt* maize has the potential to reduce yield variability due to lepidopteran pests. While *Bt*-maize can be an important component in effectively managing important insect-pests affecting maize yields in sub-Saharan Africa, conventional pest management practices should be maintained in order to avoid reliance on a single control strategy. *Bt-*maize, as a highly specific and efficient stem borer control measure, has great potential to enable smallholder maize farmers in Kenya to produce high-quality grain with increased yield, reduced dependence on insecticides, and improved food security.

## Figures and Tables

**Table 1:** List of Bt hybrids, non-Bt near isogenic hybrids and commercial checks.

Entry code (Bt hybrids)	Entry code (non-Bt hybrids, near isogenic hybrids)	Commercial checks (Non-*Bt* hybrids)
Hybrid1-MON810 (Entry 1)	Hybrid 1 (Entry 2)	KH414-3 (Entry 15)
Hybrid 2-MON810 (Entry 3)	Hybrid 2 (Entry 4)	H516 (Entry 16)
Hybrid 3-MON810 (Entry 5)	Hybrid 3 (Entry 6)	WH505 (Entry 17)
Hybrid 4-MON810 (Entry 7)	Hybrid 4 (Entry 8)	DK8053 (Entry 18)
Hybrid 5-MON810 (Entry 9)	Hybrid 5 (Entry 10)	
Hybrid 6-MON810 (Entry 11)	Hybrid 6 (Entry 12)	
Hybrid 7-MON810 (Entry 13)	Hybrid 7 (Entry 14)	

**Table 2:** Mean leaf damage of *Bt* hybrids and their near isogenic hybrids caused by *C. partellus* at Kiboko, Kenya, in three seasons. Plants were artificially infested with *C. partellus* 14 and 24 days after planting; leaf damage-1 was assessed 24 days after planting and leaf damage-2 was assessed 39 days after planting based on 1–9 visual scale. Pair mean values with the same letter in a column are not significantly different at *P* = 0.05 using Kruskall-Wallis non-parametric analyses (±indicates standard error of the mean).

Entry	Leaf damage 1 (±SE)			Leaf damage 2 (±SE)		
	Season 1	Season 2	Season 3	Season 1	Season 2	Season 3
1 (Bt)	3.2 ± 0.7a	1.3 ± 0.1a	1.0 ± 0.0a	5.1 + 0.9a	2.9 ± 0.0a	1.1 ± 0.0a
2 (Non-Bt)	4.5 ± 1.2a	4.8 ± 0.4b	3.7 ± 0.2b	4.9 + 1.0b	4.7 ± 0.1b	5.0 ± 0.1b
3 (Bt)	4.7 ± 0.4a	1.4 ± 0.1a	1.0 ± 0.0a	5.0 ± 0.4a	3.1 ± 0.2a	1.1 ± 0.0a
4 (Non-Bt)	2.5 ± 0.5b	6.6 ± 0.5b	4.5 ± 0.2b	3.7 ± 0.3b	5.3 ± 0.1b	5.3 ± 0.1b
5 (Bt)	4.2 ± 0.4a	1.7 ± 0.1a	1.2 ± 0.2a	4.8 ± 0.3a	3.4 ± 0.1a	1.5 ± 0.1a
6 (Non-Bt)	5 ± 0.4a	5.6 ± 0.2b	4.2 ± 0.4b	2.5 ± 0.6b	4.6 ± 0.3b	5.9 ± 0.3b
7 (Bt)	3.7 ± 0.7a	1.4 ± 0.1a	1.2 ± 0.2a	3.7 ± 1.0a	3.2 ± 0.0a	1.1 ± 0.0a
8 (Non-Bt)	3.0 ± 0.5a	6.1 ± 0.4b	5.0 ± 0.4b	5.0 ± 0.9a	5.3 ± 0.2b	5.3 ± 0.6b
9 (Bt)	5.0 ± 0.7a	1.5 ± 0.1a	1.7 ± 0.2a	4.6 ± 0.7a	3.0 ± 0.2a	1.2 ± 0.0a
10 (Non-Bt)	3.2 ± 0.8a	5.3 ± 0.3b	3.7 ± 0.2b	4.1 ± 0.8a	4.4 ± 0.1b	5.1 ± 0.5b
11 (Bt)	2.7 ± 0.8a	1.8 ± 0.3a	1.2 ± 0.2a	3.6 ± 0.9a	3.5 ± 0.1a	1.1 ± 0.1a
12 (Non-Bt)	3.7 ± 1.0a	6.1 ± 0.1b	3.7 ± 0.2b	3.6 ± 1.0a	4.9 ± 0.1b	5.0 ± 0.4b
13 (Bt)	4.5 ± 0.6a	1.4 ± 0.1a	1.0 ± 0.0a	3.0 ± 0.9a	3.1 ± 0.1a	1.3 ± 0.1a
14 (Non-Bt)	3.7 ± 0.7a	6.0 ± 0.2b	3.7 ± 0.2b	4.8 ± 0.4a	5.3 ± 0.3b	5.1 ± 0.4b

**Table 3:** Mean exit holes of *Bt* hybrids and their near isogenic hybrids caused by *C. partellus* at Kiboko, Kenya, in three seasons. Entries with odd numbers have *Bt* gene and those with even numbers do not have *Bt* gene (near isogenic hybrids). Plants artificially infested with *C. partellus* 14 and 24 days after planting; exit holes were assessed at harvest. Pair mean values with the same letter in a column are not significantly different at *P* = 0.05 using *t*-tests.

Exit holes per plant			
Entry	Season 1	Season 2	Season 3
1 (Bt)	0.1 ± 0.1*a	0.5 ± 0.3a	0.0 ± 0.0a
2 (Non-Bt)	1.8 ± 0.6b	3.9 ± 0.5b	2.0 ± 0.4b
3 (Bt)	0.1 ± 0.1a	1.2 ± 0.9a	0.0 ± 0.0a
4 (Non-Bt)	3.4 ± 0.7b	3.1 ± 0.9a	1.0 ± 0.2b
5 (Bt)	0.2 ± 0.2a	1.5 ± 0.8a	0.0 ± 0.0a
6 (Non-Bt)	2.9 ± 0.5b	3.9 ± 0.7b	0.6 ± 0.2b
7 (Bt)	0.9 ± 0.5a	5.7 ± 5.7a	0.0 ± 0.0a
8 (Non-Bt)	3.1 ± 0.5b	5.1 ± 0.2a	1.5 ± 0.4b
9 (Bt)	0.0 ± 0.0a	0.5 ± 0.2a	0.0 ± 0.0a
10 (Non-Bt)	1.6 ± 0.3b	3.7 ± 0.7b	1.4 ± 0.5b
11 (Bt)	1.5 ± 0.7a	8.1 ± 3.1a	0.0 ± 0.0a
12 (Non-Bt)	4.0 ± 0.3b		2.6 ± 0.6b
13 (Bt)	0.0 ± 0.0a	0.5 ± 0.2a	0.0 ± 0.0a
14 (Non-Bt)	2.2 ± 0.2b	3.3 ± 0.5b	1.2 ± 0.2b

* = ± Standard error of the mean.

**Table 4:** Mean percent tunnel length of *Bt* hybrids and their near isogenic hybrids caused by *C. partellus* at Kiboko, Kenya, in three seasons. Entries with odd numbers have *Bt* gene and those with even numbers do not have *Bt* gene (near isogenic hybrids). Plants were artificially infested with *C. partellus* 14 and 24 days after planting; tunnel length was assessed at harvest. Pair mean values with the same letter in a column are not significantly different at *P* = 0.05 using *t*-tests (±indicates standard error of the mean).

Tunnel length (%)			
Entry	Season 1	Season 2	Season 3
1 (Bt)	0.0 ± 0.0a	0.0 ± 0.0a	0.0 ± 0.0a
2 (Non-Bt)	4.4 ± 0.7b	3.5 ± 0.3b	2.6 ± 0.6b
3 (Bt)	0.1 ± 0.1a	0.1 ± 0.0a	0.0 ± 0.0a
4 (Non-Bt)	4.7 ± 0.47b	2.6 ± 1.0b	1.6 ± 0.3b
5 (Bt)	0.3 ± 0.3a	0.0 ± 0.0a	0.0 ± 0.0a
6 (Non-Bt)	3.5 ± 1.6b	6.3 ± 1.0b	1.6 ± 0.3b
7 (Bt)	0.7 ± 0.5a	0.3 ± 0.3a	0.0 ± 0.0a
8 (Non-Bt)	4.5 ± 0.9b	5.5 ± 0.5b	2.9 ± 0.8b
9 (Bt)	1.4 ± 1.4a	0.0 ± 0.0a	0.2 ± 0.1a
10 (Non-Bt)	2.8 ± 0.2a	2.8 ± 0.8b	2 ± 0.5b
11 (Bt)	1.5 ± 0.6a	0.0 ± 0.0a	0.2 ± 0.1a
12 (Non-Bt)	7.0 ± 0.4b	7.9 ± 2.0b	4.1 ± 0.5b
13 (Bt)	1.3 ± 1.3a	0.0 ± 0.0a	0.0 ± 0.0a
14 (Non-Bt)	3.1 ± 0.4a	2.6 ± 0.1b	2.2 ± 0.4b

**Table 5:** Mean grain yield (t ha^−1^) of *Bt* hybrids and their near isogenic hybrids caused by *C. partellus* at Kiboko, Kenya, in three seasons. Entries with odd numbers have *Bt* gene and those with even numbers do not have *Bt* gene (near isogenic hybrids). Plants were artificially infested with *C. partellus* 14 and 24 days after planting; yield was assessed at harvest. Pair mean values with the same letter in a column are not significantly different at *P* = 0.05 using *t*-tests (±indicates standard error of the mean).

Yield ha^−2^			
Entry	Season 1	Season 2	Season 3
1 (Bt)	6.7 ± 0.7a	12.9 ± 0.5a	11.2 ± 0.3a
2 (Non-Bt)	5.2 ± 0.6a	10.5 ± 0.7b	7.1 ± 0.7b
3 (Bt)	7.8 ± 0.1a	14.7 ± 1.5a	11.6 ± 0.7a
4 (Non-Bt)	7.2 ± 0.6a	6.8 ± 2.0b	9.2 ± 0.9b
5 (Bt)	7.1 ± 0.3a	12.7 ± 1.1a	10.5 ± 0.8a
6 (Non-Bt)	6.2 ± 0.3a	6.1 ± 1.2b	4.8 ± 0.5b
7 (Bt)	5.9 ± 0.6a	11.5 ± 1.0a	11.7 ± 0.2a
8 (Non-Bt)	6.6 ± 0.4a	6.9 ± 0.2b	6.9 ± 1.1b
9 (Bt)	5.2 ± 0.2a	10.7 ± 0.6a	10.0 ± 0.4a
10 (Non-Bt)	4.5 ± 0.4a	6.1 ± 1.3b	5.8 ± 1.3b
11 (Bt)	4.2 ± 0.3a	5.2 ± 0.8a	10.3 ± 0.4a
12 (Non-Bt)	3.7 ± 0.5a	4.0 ± 0.7a	5.9 ± 0.4b
13 (Bt)	7.7 ± 1.1a	14.2 ± 0.9a	11.8 ± 0.6a
14 (Non-Bt)	6.2 ± 0.6a	10.3 ± 1.7b	8.1 ± 0.5a

**Table 6:** Mean of entry by season interactions and main effects amongst three sets of entries (*Bt* hybrids, non-*Bt* near isogenic hybrids and non-*Bt* commercial checks) averaged across season and across all *Bt* hybrids, non-*Bt* hybrids and across the commercial checks. Mean values with the same letter in a column are not significantly different using the least significance difference (LSD) at P = 0.05.

Season	Entry^∗^	Grain yield (t ha^−1^) (±SEM)	Number holes/plant (±SEM)	% Stem height tunnelled (±SEM)
1	Bt	6.4 ± 0.4ab	0.4 ± 0.3a	0.7 ± 0.3a
	Non-Bt	5.7 ± 0.4ab	2.7 ± 0.3c	3.8 ± 0.3c
	Checks	5.0 ± 0.5a	2.8 ± 0.5c	5.0 ± 0.4d
2	Bt	11.7 ± 0.4d	1.7 ± 0.3b	0.1 ± 0.3a
	Non-Bt	7.2 ± 0.4bc	4.0 ± 0.3d	3.9 ± 0.3c
	Checks	5.9 ± 0.5ab	4.9 ± 0.5d	4.8 ± 0.4d
3	Bt	11.0 ± 0.4d	0.03 ± 0.3a	0.07 ± 0.3a
	Non-Bt	6.9 ± 0.4ab	1.5 ± 0.3b	2.1 ± 0.3b
	Checks	7.0 ± 0.5bc	2.1 ± 0.5b	3.4 ± 0.4c
Mean entry	Bt	9.7 ± 0.2c	0.7 ± 0.2a	0.3 ± 0.1a
	Non-Bt	6.9 ± 0.2b	2.8 ± 0.2b	3.7 ± 0.1b
	Checks	6.0 ± 0.3a	3.3 ± 0.2c	4.7 ± 0.2c
Mean season	1	5.8 ± 0.2a	2.0 ± 0.2b	3.3 ± 0.2b
	2	8.3 ± 0.2b	3.6 ± 0.2c	3.1 ± 0.2b
	3	8.4 ± 0.2b	1.2 ± 0.2a	2.3 ± 0.2a

*Entry Bt = Bt hybrids; Non-Bt = Non-Bt near isogenic hybrids; Checks = Commercial checks.

**Table 7:** Mean leaf damage of *Bt* hybrids and their near isogenic hybrids caused by *B. fusca* in biosafety greenhouse under artificial infestation twice with ten neonates per plant 21 and 31 days after planting. Leaf damage was assessed twice, 31 and 41 days after planting using the 1–9 visual scoring scale. Pair mean values with the same letter in a column are not significantly different at *P* = 0.05 using Kruskall-Wallis non-parametric analyses (±indicates standard error of the mean).

Entry	Leaf damage 1	Leaf damage 2
1 (Bt)	2.5 ± 0.1a	3.7 ± 0.2a
2 (Non-Bt)	1.5 ± 0.1a	4.2 ± 0.2a
3 (Bt)	2.5 ± 0.2a	2.2 ± 0.2a
4 (Non-Bt)	4.7 ± 0.8b	4.2 ± 0.7b
5 (Bt)	2.2 ± 0.2a	2.0 ± 0.1a
6 (Non-Bt)	3.2 ± 0.4b	3.0 ± 0.4b
7 (Bt)	2.5 ± 0.2a	1.7 ± 0.2a
8 (Non-Bt)	4.0 ± 0.4b	4.7 ± 0.4b
9 (Bt)	2.2 ± 0.2a	2.0 ± 0.1a
10 (Non-Bt)	4.5 ± 0.8b	4.0 ± 0.2b
11 (Bt)	2.7 ± 0.4a	2.0 ± 0.1a
12 (Non-Bt)	4.2 ± 0.2b	5.0 ± 0.2b
13 (Bt)	2.0 ± 0.1a	2.0 ± 0.1a
14 (Non-Bt)	3.2 ± 0.2b	3.0 ± 0.1a
